# Multi-Scale Temporal-Dependency Learning for Single-Sensor Gas Classification

**DOI:** 10.3390/s26185763

**Published:** 2026-09-10

**Authors:** Longlong Yu, Gen Li, Zhicheng Cai

**Affiliations:** 1 College of Computer Science, Chengdu University, Chengdu 610106, China; yulonglong@cdu.edu.cn (L.Y.); ligen@cdu.edu.cn (G.L.); 2 Department of Semiconductor System Engineering, Sejong University, Seoul 05006, Republic of Korea

**Keywords:** gas classification, metal oxide semiconductor sensor, temporal-dependency learning, reservoir computing, correlation analysis, multi-scale representation

## Abstract

Single-sensor gas classification remains challenging because metal oxide semiconductor sensors exhibit limited selectivity and different gases can produce similar response patterns. This paper proposes a multi-scale temporal-dependency-learning framework that combines reservoir-based multi-scale dynamic learning (MsDL) with lagged-correlation features. To reduce information leakage caused by window construction, each continuous gas-response sequence is divided chronologically into training, validation, and test blocks before window extraction, and 2000 raw samples are excluded between adjacent subsets. The primary evaluation uses non-overlapping windows and fits all data-dependent preprocessing using the training subset only. On a dataset containing seven volatile organic compounds measured using a single sensor, the proposed MsDL+Corr representation with a random-forest classifier achieves a test accuracy of 92.14% and a macro-F1 score of 92.08%. Across ten random-forest seeds, the accuracy is 92.43% ± 0.37%. The corresponding micro-average one-vs-rest AUC is 0.9948 (99.48%); this threshold-independent ranking metric is distinct from classification accuracy. Feature ablation shows that combining MsDL and correlation features improves the macro-F1 score over either branch alone. These results demonstrate that temporal-dependency representations provide useful discriminative information under a conservative temporally separated evaluation protocol.

## 1. Introduction

Metal oxide semiconductor (MOS) gas sensors have been widely used in environmental monitoring, industrial safety, food quality assessment, and healthcare applications because of their low cost, high sensitivity, fast response, and ease of miniaturization [1,2,3]. Despite these advantages, accurate gas classification remains challenging because MOS sensors inherently suffer from cross-sensitivity, allowing different gases to produce similar response patterns [4]. To improve gas discriminability, electronic-nose systems based on sensor arrays have been extensively investigated, where responses from multiple sensing elements are combined with pattern-recognition algorithms for gas identification [5]. However, the use of multiple sensors increases hardware complexity, calibration requirements, power consumption, and deployment cost, limiting their applicability in compact and resource-constrained scenarios. Consequently, developing effective classification methods using a single sensor has attracted increasing attention for portable and low-cost gas-sensing systems [6].

Existing studies have mainly improved gas classification by combining sensor responses with feature engineering, machine learning, and deep learning models [7]. Early approaches usually extracted statistical or steady-state descriptors from gas-response curves and used pattern-recognition algorithms for classification, while more recent studies have employed models such as SVM, MLP, random forest, and convolutional or recurrent neural networks to learn discriminative patterns from sensor time series [4,8]. For example, Zhou and Liu developed a convolutional long short-term-memory network for early-stage gas identification using sensor-array time-series data, showing that temporal information can improve recognition accuracy compared with MLP and SVM baselines [8]. Recent single-sensor studies have further attempted to compensate for limited sensor selectivity through special excitation strategies and deep learning models, such as time-variant illumination or pulse-driven MEMS sensing, which generate richer transient responses for gas identification [9,10]. These studies suggest that the information contained in the response process is useful for gas classification, but most existing methods still rely on either learned representations from raw responses or device-level modulation strategies, rather than explicitly characterizing the temporal-dependency structure of the response signal.

Most existing studies distinguish gases according to response amplitudes, statistical descriptors, or features learned from sensor signals. These representations focus on the magnitude and shape of sensor responses. However, gas sensing is a dynamic process rather than a collection of independent observations. The response of a sensor continuously evolves after gas exposure, and the evolution pattern varies across gas species. Consequently, two gases may exhibit similar response amplitudes while following different temporal trajectories. This suggests that discriminative information may exist not only in terms of response values but also in the dependency relationships formed during the response process. Although time-series models have been introduced into gas sensing, temporal dependencies are usually learned implicitly and rarely analyzed as an independent source of discriminative information. Therefore, how to characterize temporal-dependency structures and exploit them for single-sensor gas classification remains an open problem. The above observations reveal several challenges in single-sensor gas classification:

- Existing methods primarily focus on response magnitudes and local signal variations, while temporal-dependency structures embedded in gas-response processes remain underexplored.
- Gas responses evolve across multiple temporal scales, making it difficult to characterize both short-range and long-range dependencies using a single representation.
- The relationship between temporal-dependency patterns and gas discriminability has not been systematically investigated in single-sensor gas classification.

To address these challenges, we propose a multi-scale temporal-dependency-learning framework for single-sensor gas classification. The central idea is to characterize gas responses from the perspective of temporal dependencies rather than relying solely on response magnitudes. Specifically, sensor responses are analyzed at multiple temporal intervals to capture dependency patterns across different time scales. A reservoir-based dynamic-learning module is employed to describe the evolution of response dynamics, while correlation analysis is introduced to quantify statistical dependencies within the response process. The resulting representations provide complementary information from both dynamic and statistical perspectives. By integrating these representations, the proposed framework constructs a multi-scale temporal-dependency descriptor for gas classification. In this way, temporal-dependency structures can be explicitly modeled and utilized to improve gas discriminability.

The main contributions of this paper are summarized as follows:

- We investigate temporal-dependency structures in gas-response signals and examine their role in distinguishing different gas species.
- We propose a multi-scale temporal-dependency-learning framework that characterizes gas responses from both dynamic evolution and statistical-dependency perspectives.
- Extensive experiments on seven volatile organic compounds demonstrate that temporal-dependency structures provide discriminative information beyond conventional response-based features and improve gas-classification performance.

The organization of this paper is as follows. Section 2 reviews related studies on MOS gas classification, single-sensor gas identification, and temporal feature learning. Section 3 presents the proposed multi-scale temporal-dependency-learning framework. Section 4 describes the experimental setup and discusses the results. Section 5 concludes this paper and outlines future work.

## 2. Related Work

### 2.1. Machine Learning for MOS Gas Classification

Machine learning has been widely used to improve gas classification in MOS sensing systems. Traditional approaches usually extract handcrafted features from sensor-response curves, such as steady-state values, response slopes, recovery characteristics, and statistical descriptors. These features are then combined with classifiers such as SVM, KNN, decision trees, random forests, and neural networks for gas identification [4,5].

Recent studies have further introduced deep learning and hybrid learning models into gas sensing. CNN, RNN, LSTM, and transformer-based models have been used to learn nonlinear patterns from sensor time series. Zhou and Liu used a convolutional LSTM model for early-stage gas identification and showed that temporal information can improve classification performance compared with MLP and SVM baselines [8]. More recent studies also indicate that machine learning can improve gas classification in sensor arrays and VOC-detection tasks, especially when the sensor responses contain complex nonlinear and cross-sensitive patterns [11,12].

At the same time, recent reviews emphasize that pattern recognition is becoming a key component of intelligent gas-sensing systems [4,12]. These studies show that machine learning can improve recognition accuracy by extracting discriminative information from gas-response data. However, most methods still focus on response amplitudes, engineered statistical features, or latent representations learned from raw signals. The temporal-dependency structure embedded in the response process is rarely analyzed as an explicit and independent source of discriminative information.

### 2.2. Single-Sensor Gas Classification

Sensor arrays are commonly used to improve gas selectivity, but they also increase system size, calibration cost, power consumption, and signal-processing complexity. For portable gas-sensing systems, single-sensor classification is, therefore, an attractive direction. The key problem is how to obtain sufficient discriminative information from only one sensing element.

Recent studies have mainly improved single-sensor classification by enriching the response process. Mei et al. developed a single-sensor VOC recognition system using a multi-task deep learning model [13]. Kim et al. developed an ultralow-power single-sensor e-nose system based on duty cycling and deep learning for real-time gas identification [14]. Wawrzyniak et al. used a single thermally modulated MOS gas sensor with a PCA–ANN model to quantify volatile compounds in mixtures [15].

These studies show that single-sensor gas classification is feasible when richer dynamic responses are introduced through temperature modulation or duty-cycling operation. However, most existing methods improve discriminability by changing the sensor-operation mode. Fewer studies focus on extracting temporal-dependency information directly from the original response sequence. This leaves room for signal-level methods that improve single-sensor gas classification without relying heavily on additional hardware modulation.

### 2.3. Temporal-Dependency Learning in Sensor Signals

Sensor responses are typical time-series signals whose resistance continuously evolves during gas adsorption and desorption. Therefore, the temporal order of response points contains valuable information for gas identification. Ignoring these temporal dynamics may lead to the loss of discriminative characteristics embedded in the transient sensing process.

Temporal learning methods have been widely used to model sequential data. Recurrent neural networks, LSTM-based models, temporal convolutional networks, and transformer architectures can effectively capture short- and long-range temporal dependencies. In gas sensing, these models have demonstrated superior performance to conventional static feature-based methods by directly learning dynamic representations from sensor-response curves. Recent studies have further shown that transient response signals contain richer discriminative information than steady-state responses alone. For example, Jiang et al. proposed a chip-based portable electronic nose that combines optimized temporal response slices with an LSTM–CNN model for rapid essence evaluation [16]. Lee et al. employed temperature-modulated operation of a single MOS gas sensor together with a deep convolutional neural network to simultaneously identify gases and estimate their concentrations [17]. Dridi et al. systematically compared multiple feature-extraction strategies based on adsorption–desorption dynamics, including kinetic descriptors, curve-fitting parameters, and frequency-domain features, demonstrating that transient response characteristics provide substantially more discriminative information than equilibrium response values alone [18].

Reservoir computing provides another effective framework for temporal representation learning. Instead of directly optimizing recurrent parameters, reservoir computing projects the input sequence into a high-dimensional dynamic state space, where the state trajectory naturally preserves temporal dependencies with low computational cost [19,20]. Recent studies have further demonstrated the effectiveness of reservoir-based models for both regular and irregular time-series classification, making them particularly attractive for resource-constrained sensing applications [21].

Although existing studies have successfully exploited dynamic sensor responses, most methods rely on temporal slices, deep neural network architectures, or manually designed kinetic descriptors. Explicit modeling of temporal-dependency relationships across multiple temporal lags has received much less attention, especially for single-sensor gas classification. Moreover, statistical-dependency information embedded in the response evolution is rarely integrated with dynamic temporal representations within a unified framework. Therefore, the novelty of the present study does not lie simply in using dynamic response curves. Instead, it lies in integrating skip-dependent reservoir dynamics with lagged-correlation features extracted from the original response sequence and its first-order difference. By jointly modeling dynamic and statistical temporal dependencies, the proposed framework provides a comprehensive temporal representation for single-sensor VOC classification without requiring additional hardware modulation or a deep neural network architecture.

## 3. Methodology

### 3.1. Framework Overview

The proposed framework aims to characterize temporal-dependency structures embedded in single-sensor gas-response signals.

Starting from each response window, the method forms a three-channel input comprising the raw response, its first difference, and its second difference. Temporal scale is introduced inside the recurrent update through skip values k∈{1,2,4,8,16,32}; the input sequence is not divided into independently sampled sub-sequences.

### 3.2. Feature Extraction

Two complementary feature branches are employed. The MsDL branch projects the three-channel input into a 50-dimensional reservoir state space and learns a scale-specific linear readout. The correlation branch directly quantifies lagged statistical dependence in the standardized response and first-difference sequences.

For skip *k*, the reservoir state is updated according to

(1) $$h_{k}(t)=\tanh\left(\mu_{k}W_{0}h_{k}\left(t-k\right)+W_{in}x\left(t\right)\right),$$

where $x\left(t\right)={\left[x\left(t\right),\Delta x\left(t\right),\Delta^{2}x\left(t\right)\right]}^{T}$, $W_{in}$ is the input matrix, and $W_{0}$ is a shared dense recurrent matrix normalized to unit spectral radius. States preceding the available history are initialized to zero. The entries of $W_{in}$ are drawn uniformly from [−0.5,0.5], and the entries of the unnormalized recurrent matrix are drawn uniformly from [−1,1], using random seed 42. The skip-specific multipliers are $\mu_{k}=\left\{0.30,0.50,0.70,0.80,0.90,0.95\right\}$ for k={1,2,4,8,16,32}, respectively. Thus, each scale changes both the recurrent delay and the effective spectral radius.

In parallel, the statistical branch z-score standardizes each response window and calculates Pearson correlations between x(1:T−k) and x(1+k:T) for the same six lag values. The procedure is repeated for the first-difference sequence. This yields 12 correlation features—two for each lag—and does not involve correlations among multiple sensors.

The complete MsDL descriptor and the 12 correlation features are concatenated into a unified representation. All feature-wise standardization parameters are subsequently estimated from the training subset only and applied unchanged to the validation and test subsets.

### 3.3. Skip-Dependent Temporal-Scale Construction

Gas sensing is a dynamic process in which sensor responses evolve continuously after gas exposure. Dependencies at small lags mainly reflect local variations, whereas larger lags can retain information about slower response trends. The proposed construction, therefore, evaluates the fixed input window at six recurrent skips rather than downsampling it into shorter sequences.

**Figure 1:**
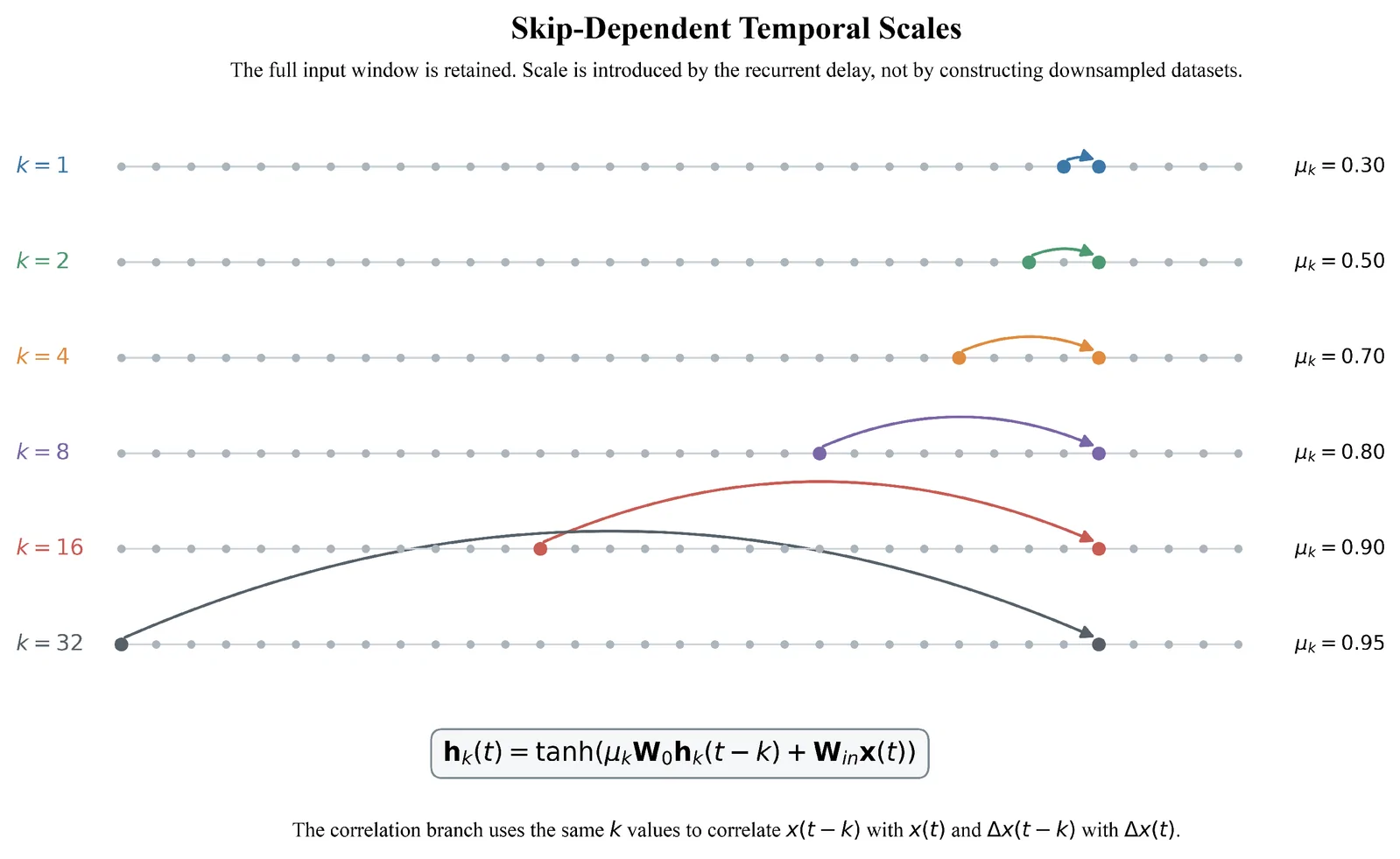
Schematic illustration of skip-dependent temporal scales. The labels denote recurrent delays *k*, not independently downsampled datasets. Smaller skips emphasize local evolution, whereas larger skips link reservoir states over longer temporal intervals.

Figure 1 illustrates the temporal-scale construction.

The same six values are also used as lags in the correlation branch so that the dynamic and statistical descriptors cover corresponding temporal intervals.

### 3.4. Reservoir-Based Dynamic Representation Learning

Temporal dependencies are reflected not only by individual response values but also by the evolution of response trajectories over time. For every skip, the reservoir-state matrix is converted into a fixed-length descriptor by fitting a ridge readout to predict the input vector *k* samples ahead.

Let $H_{k}$ collect the reservoir states and let $Y_{k}={\left[x\left(1+k\right),\ldots ,x\left(T\right)\right]}^{T}$ denote the aligned future targets. The scale-specific ridge readout is obtained as

(2) $$B_{k}=\arg\min_{B}{∥H_{k}B^{T}-Y_{k}∥}_{F}^{2}+\alpha {∥B∥}_{F}^{2},\;\alpha =1.0,$$

with no intercept. For three input channels and 50 reservoir units, $B_{k}$ contains 150 coefficients. These coefficients are vectorized and concatenated with the readout mean squared error, giving 151 features per skip. Six skips, therefore, produce 906 MsDL features; concatenation with the 12 lagged-correlation features gives a final 918-dimensional representation.

### 3.5. Feature Fusion and Classification

The complete MsDL descriptor and correlation descriptor are combined as

(3) $$F=\left[f_{\mathrm{MsDL}},f_{\mathrm{Corr}}\right].$$

The resulting vector contains complementary information from dynamic prediction and lagged statistical dependence. Before classifier fitting, every feature is standardized using the training-set mean and standard deviation; transformed values are clipped to [−10,10] for numerical robustness. The fitted transformation is reused without refitting on the validation and test data.

The fused feature vector is subsequently used for gas classification. Random Forest (RF) is used as the prespecified primary classifier. Support Vector Machine (SVM), Logistic Regression (LR), and K-Nearest Neighbor (KNN) are evaluated as classifier controls using the same features and temporal data partitions.

For each classifier, the fused feature vector F is used as the input, and the corresponding gas category is used as the target label. Classification performance is evaluated using accuracy, macro-precision, macro-recall, macro-F1, weighted F1, and one-vs-rest AUC. The experiments presented in Section 4 use identical chronologically separated subsets for all compared configurations.

## 4. Experimental Results

### 4.1. Dataset and Leakage-Controlled Validation Protocol

The dataset was obtained using a single chemiresistive MOS gas sensor based on SnO_2_ nanowires. The sensor resistance was continuously recorded during exposure to seven VOCs: ethanol, acetone, methanol, toluene, xylene, benzene, and triethylamine (TEA). All measurements were conducted at an operating temperature of 275 °C using synthetic air as the carrier gas, with a total flow rate of 100 sccm and a relative humidity of 0.5%. The concentration of each target gas was fixed at 20 ppm. Each processed gas record contained 150,000 resistance samples without missing values. The present study reanalyzed archived processed records and did not involve new sensor fabrication, chemical preparation, or data acquisition; manufacturer information for the sensor, gases, and measurement system was not retained in the source files. All analyses were performed using Python 3.10.16, NumPy 1.26.4, pandas 2.2.3, and scikit-learn 1.6.1; Matplotlib 3.10.1 was used for visualization.

**Figure 2:**
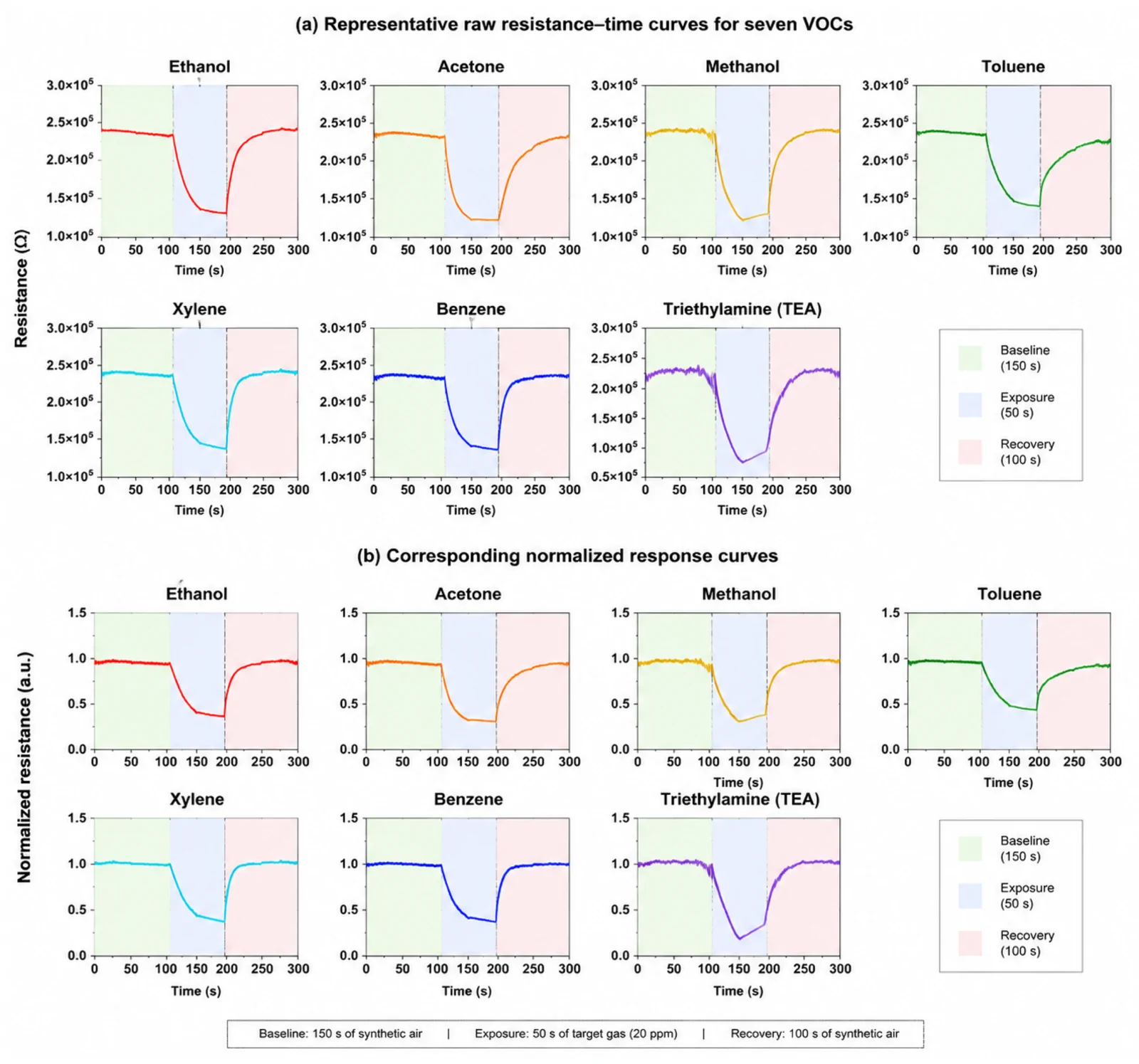
Representative sensor-response curves of the seven VOCs: (**a**) raw resistance–time curves and (**b**) normalized response curves.

Figure 2 shows representative raw and normalized response curves for the seven VOCs.

The available processed dataset stores one continuous sequence for each gas and does not retain identifiers for independently repeated exposure cycles. Consequently, treating windows extracted from the same continuous record as independent samples would overstate the effective amount of independent test information. The revised evaluation, therefore, partitions each raw sequence chronologically before any window extraction. For every gas, samples 0–87,999 form the training block, samples 90,000–117,999 form the validation block, and samples 120,000–149,999 form the test block. The intervals 88,000–89,999 and 118,000–119,999 are excluded, providing a 2000-sample purge interval between adjacent subsets. Windows are subsequently constructed separately within each subset, and no window is permitted to cross a subset boundary.

The primary evaluation uses a window length and stride of 1500 samples, producing non-overlapping windows. This yields 58 training, 18 validation, and 20 test windows per gas, corresponding to 406 training, 126 validation, and 140 test windows in total. A separate sensitivity experiment uses 25% and 50% within-subset overlap while retaining the same purged raw-data blocks. The non-overlapping protocol is used for the primary conclusions because it provides the most conservative window-level estimate.

For each segment, the MsDL input contains the raw response, its first-order difference, and its second-order difference. Six temporal skips, {1,2,4,8,16,32}, are used. The reservoir dimension is 50, the input scale is 0.5, and the ridge regularization coefficient is 1.0. The corresponding spectral radii are 0.30, 0.50, 0.70, 0.80, 0.90, and 0.95. The six MsDL branches generate 906 features, while lagged correlations of the standardized response and first-order difference generate 12 additional features. Their concatenation forms the 918-dimensional MsDL+Corr representation. The final feature scaler is fitted only on the training subset and then applied unchanged to the validation and test subsets. The dataset construction and main parameter settings are summarized in Table 1 and Table 2, respectively.

### 4.2. Influence of the Validation Protocol

To quantify the effect of temporal partitioning, the feature extractor, RF classifier, window length, and model hyperparameters were held constant while the validation protocol was changed. The legacy protocol first generated 50%-overlapping windows and then divided the resulting windows into the first 60% for training and the remaining 40% for testing. The two revised protocols partitioned the raw sequence before window extraction and used the purge intervals described above. Table 3 summarizes the test performance under the three validation protocols.

**Figure 3:**
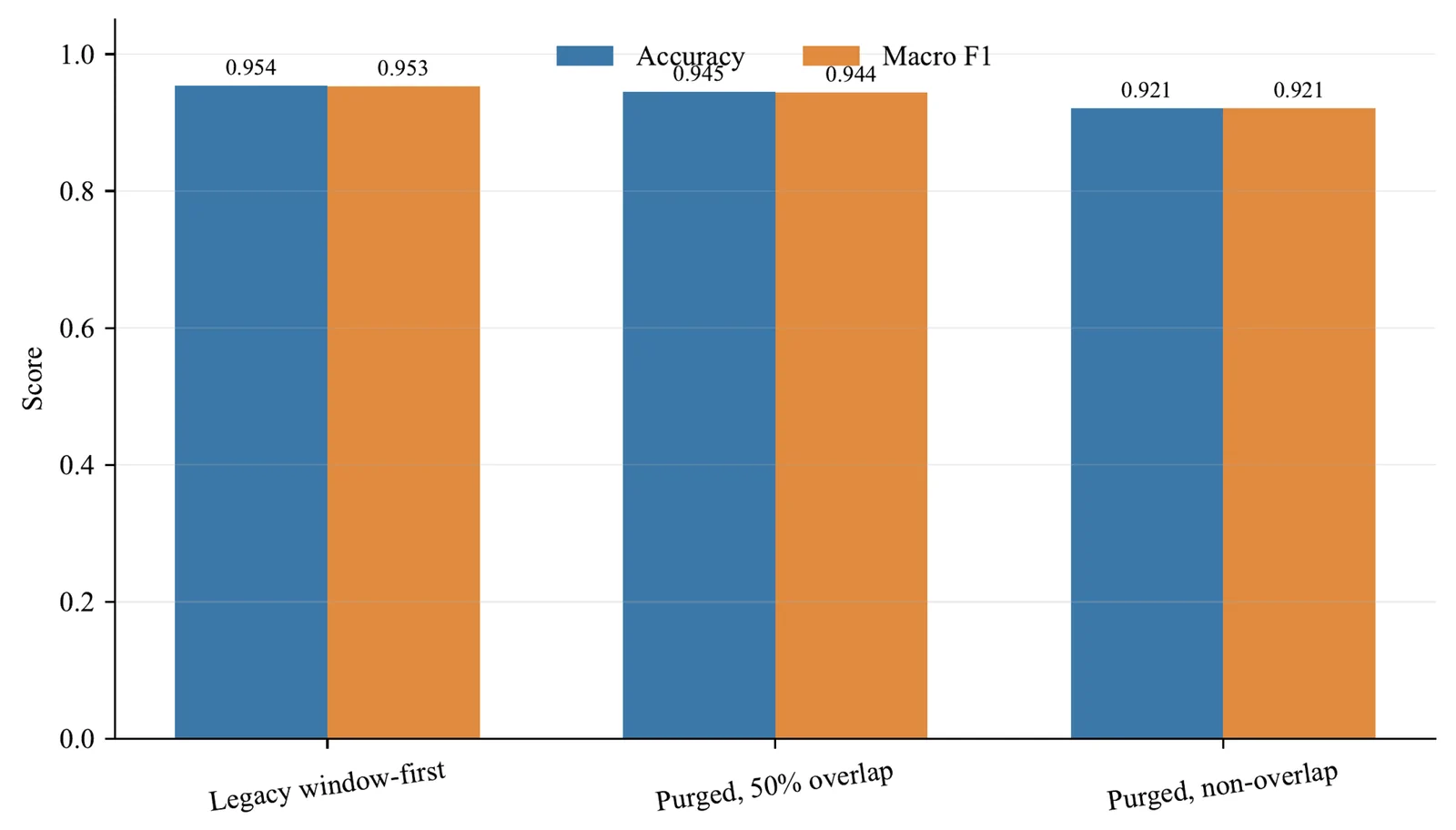
Test accuracy and macro-F1 score under the legacy window-first protocol and the two chronologically purged protocols.

As shown in Table 3 and Figure 3, the strict purged non-overlapping protocol reduces accuracy by 3.21 percentage points and macro-F1 by 3.23 percentage points relative to the legacy evaluation. This confirms that the earlier window-first protocol produced a more optimistic window-level estimate. Nevertheless, the proposed representation retains an accuracy of 92.14% under the conservative protocol. All subsequent primary results, therefore, use the purged non-overlapping split.

### 4.3. Effect of Window Size

Window length was selected using the validation subset rather than the test subset. Seven non-overlapping window lengths, 500, 750, 1000, 1250, 1500, 1750, and 2000 samples, were evaluated using the same chronologically separated raw blocks. The RF classifier and all feature-extraction parameters were held constant. Table 4 reports the validation and test results for these window lengths.

**Figure 4:**
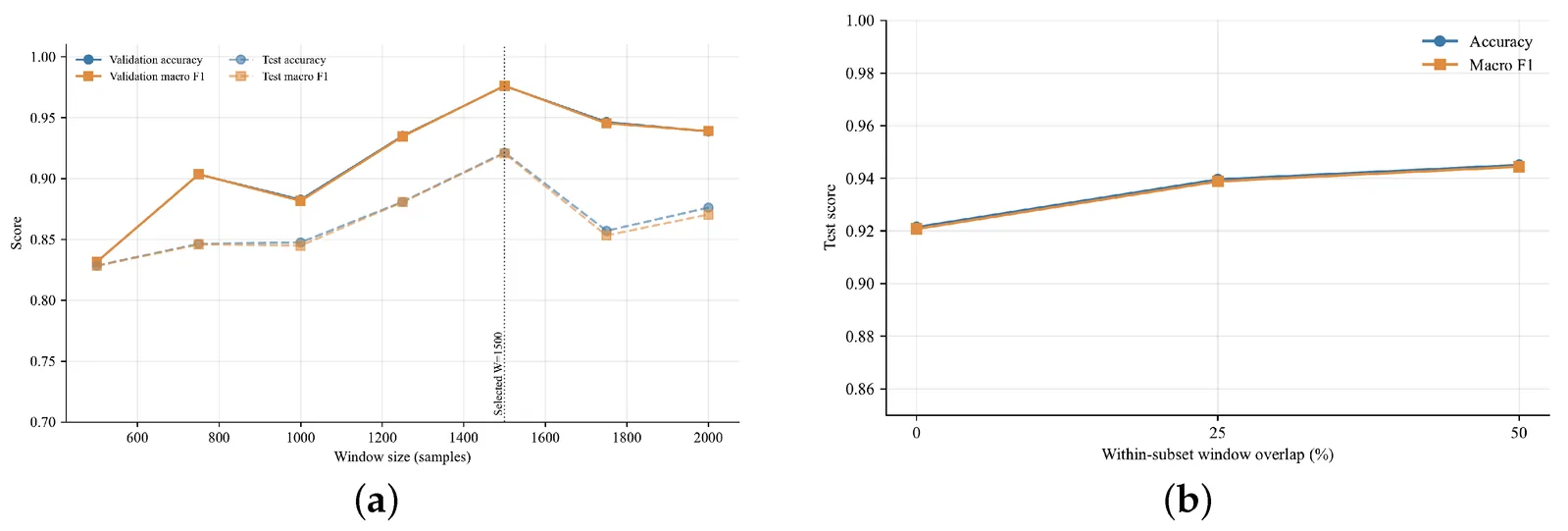
Window-construction sensitivity under the chronologically purged protocol. (**a**) Validation and test performance across non-overlapping window lengths. (**b**) Test performance across within-subset overlap ratios.

The highest validation macro-F1 score, 97.64%, is obtained at a window length of 1500 samples (Figure 4a). Shorter windows provide insufficient temporal context, whereas lengths above 1500 do not yield a further validation improvement. Accordingly, 1500 is selected before the final test evaluation and is used in all subsequent experiments.

### 4.4. Feature Ablation

The contributions of the two feature branches were evaluated under the same purged non-overlapping split using RF. The correlation-only representation contains 12 features, the MsDL representation contains 906 features, and the fused representation contains 918 features. The feature-ablation results are presented in Table 5.

**Figure 5:**
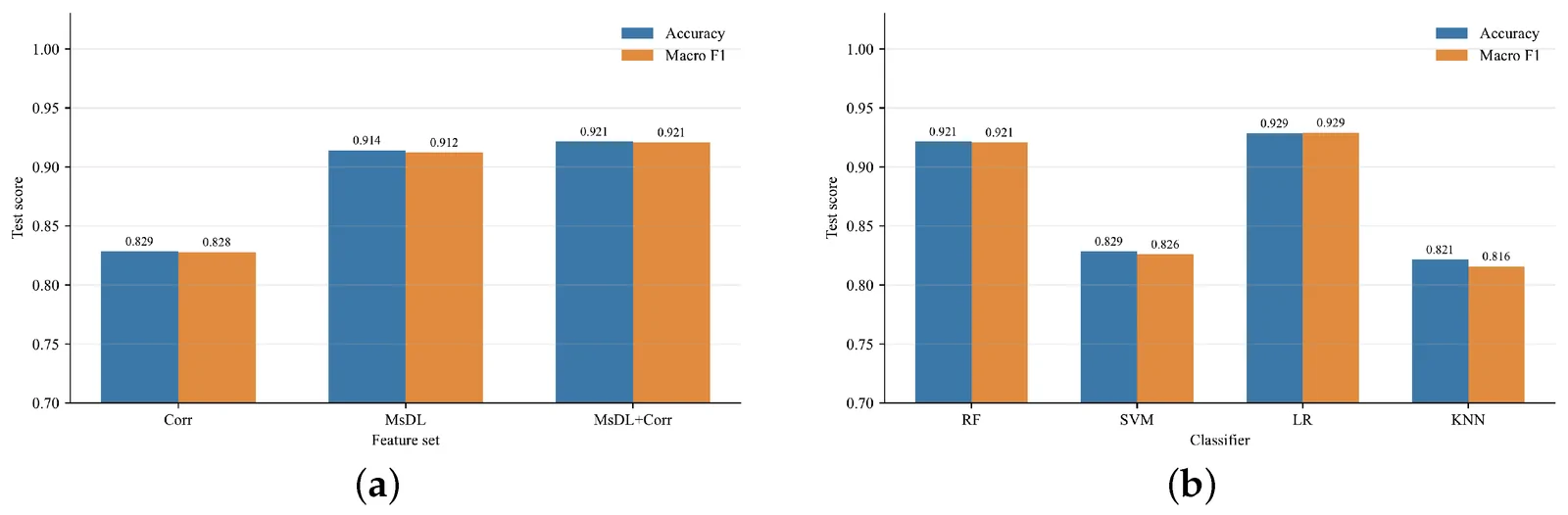
Feature and classifier controls under the purged non-overlapping protocol. (**a**) Feature-ablation comparison. (**b**) Classifier comparison using the same MsDL+Corr descriptors and temporal subsets.

MsDL provides the principal contribution, improving macro-F1 from 82.79% for correlation features to 91.21% (Figure 5a). Adding the correlation branch to MsDL further increases macro-F1 to 92.08%, an improvement of 0.86 percentage points over MsDL alone. Thus, the correlation features provide a modest but consistent complementary contribution under the strict split.

### 4.5. Classifier Comparison

The four classifiers—RF, SVM, LR, and KNN—were evaluated using the same 918-dimensional MsDL+Corr representation and purged non-overlapping subsets. RF was retained as the prespecified primary classifier; the remaining models serve as controls for classifier dependence. Table 6 compares the four classifiers on the strict test subset.

LR obtains the highest test macro-F1 score of 92.92%, exceeding RF by 0.85 percentage points (Figure 5b). RF, nevertheless, remains close to the best result and is retained as the prespecified primary classifier. SVM and KNN show substantially lower performance. These findings indicate that the proposed features are compatible with both a nonlinear ensemble and a linear-decision model, while the results do not support a claim that RF is universally superior.

### 4.6. Window-Overlap and Random-Seed Sensitivity

The influence of within-subset overlap was evaluated at a fixed window length of 1500. Overlap ratios of 0%, 25%, and 50% correspond to strides of 1500, 1125, and 750, respectively. The raw training, validation, and test blocks and purge intervals were unchanged. Table 7 summarizes the overlap-sensitivity results.

Increasing overlap raises the number of highly related test windows and is accompanied by higher window-level scores (Figure 4b). For this reason, the overlapping results are reported only as a sensitivity analysis, and the non-overlapping result remains the primary estimate.

Viewed jointly, the protocol and window analyses distinguish model performance from evaluation-design effects. The conservative protocol lowers the optimistic legacy score, validation selects a 1500-sample window, and increasing overlap raises the reported test score. These observations support retaining the purged non-overlapping setting as the primary analysis.

**Figure 6:**
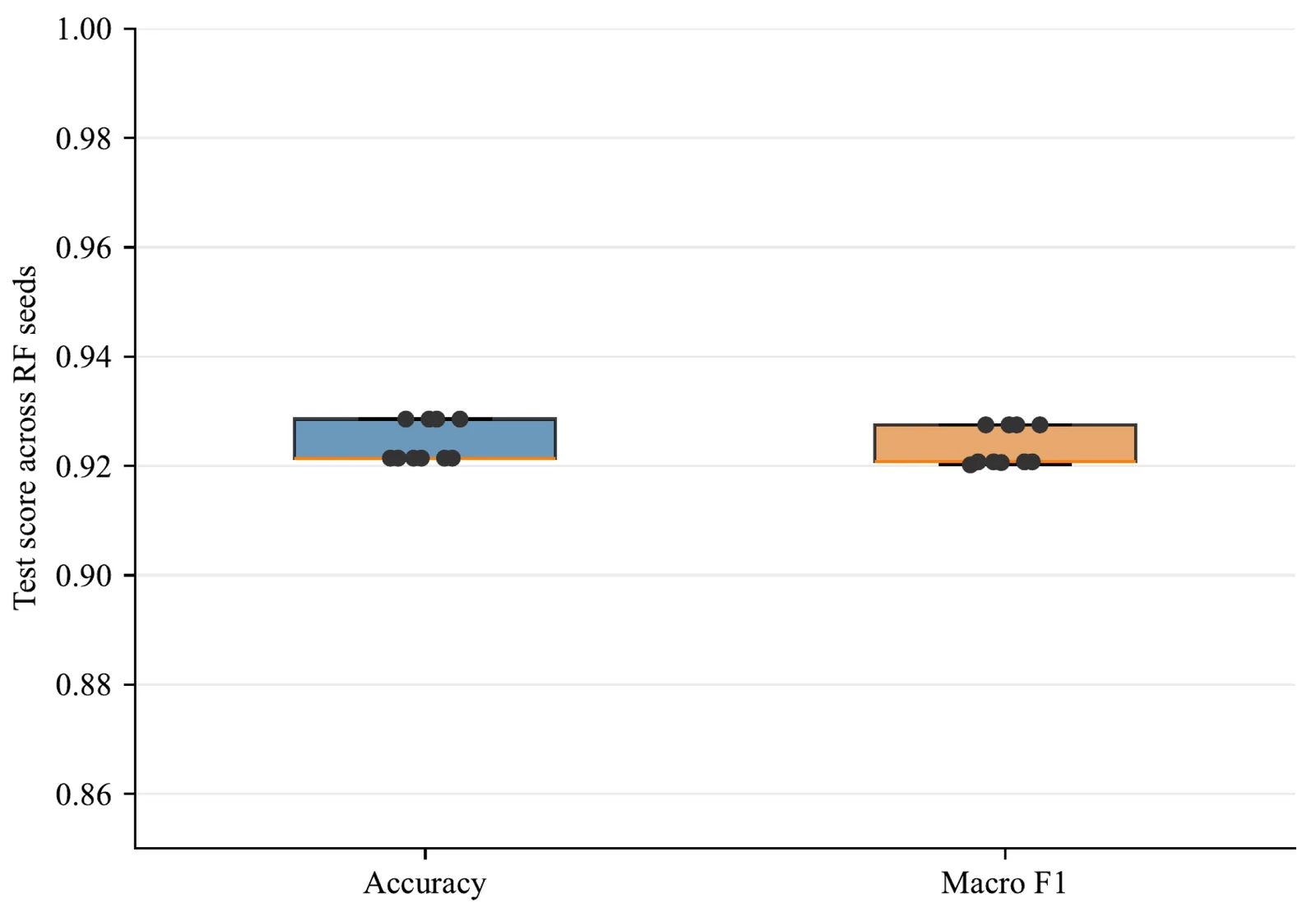
Distribution of test accuracy and macro-F1 score across ten RF random seeds.

RF initialization stability was evaluated using ten seeds: 0, 7, 21, 42, 84, 123, 2024, 3407, 7777, and 9999. Test accuracy is 92.43% ± 0.37%, with a range of 92.14–92.86%. Test macro-F1 is 92.34% ± 0.36%, with a range of 92.02–92.75% (Figure 6). The small dispersion indicates that the primary RF result is not driven by a favorable random initialization.

The paired controls show that MsDL supplies most of the discriminative gain, the correlation branch provides a smaller complementary improvement, and the fused representation also performs strongly with LR.

The narrow RF seed distribution complements the feature and classifier controls by showing that the primary RF result is stable with respect to initialization.

### 4.7. Overall Classification Performance

Under the primary purged non-overlapping protocol, acetone, benzene, and toluene are classified without errors. Ethanol achieves an F1 score of 97.56%, and methanol achieves 89.47%. The remaining errors are concentrated in TEA and xylene, whose F1 scores are 81.82% and 75.68%, respectively. Xylene recall is 70%, with 20% of xylene windows classified as TEA. TEA recall is 90%, with 10% of TEA windows classified as xylene.

**Figure 7:**
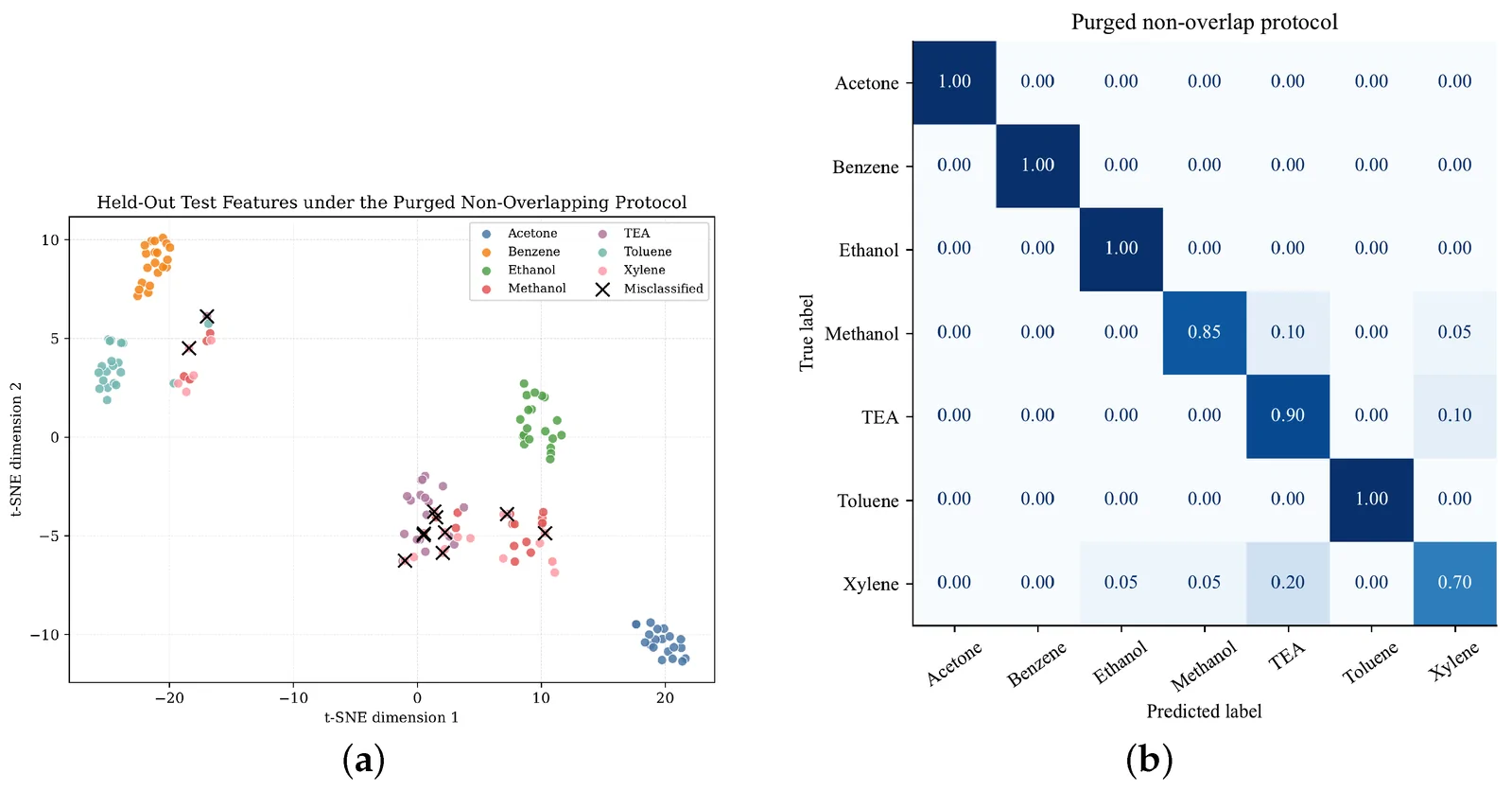
Held-out test-set structure and classification errors under the purged non-overlapping protocol. (**a**) t-SNE visualization of the 140 test windows; colors denote true classes and black crosses denote RF errors. (**b**) Row-normalized confusion matrix. The t-SNE embedding is used only for qualitative visualization.

To visualize the learned representation under the same primary protocol, the 918-dimensional fused descriptors were standardized using parameters fitted on the training subset. A 50-dimensional PCA projection fitted on training data was then applied to the held-out test descriptors before two-dimensional t-SNE embedding. Figure 7a shows compact and well-separated test clusters for acetone, benzene, ethanol, and toluene. Methanol, TEA, and xylene occupy closer regions, and most marked errors occur in these locally overlapping areas. This qualitative structure agrees with the class-wise F1 scores and does not replace the quantitative held-out evaluation.

The paired visualization and confusion matrix provide a coherent account of the remaining errors. Classes forming isolated t-SNE clusters are classified without errors, whereas the closer methanol–TEA–xylene regions contain the marked errors. In particular, the TEA–xylene proximity is reflected in their off-diagonal confusion.

**Figure 8:**
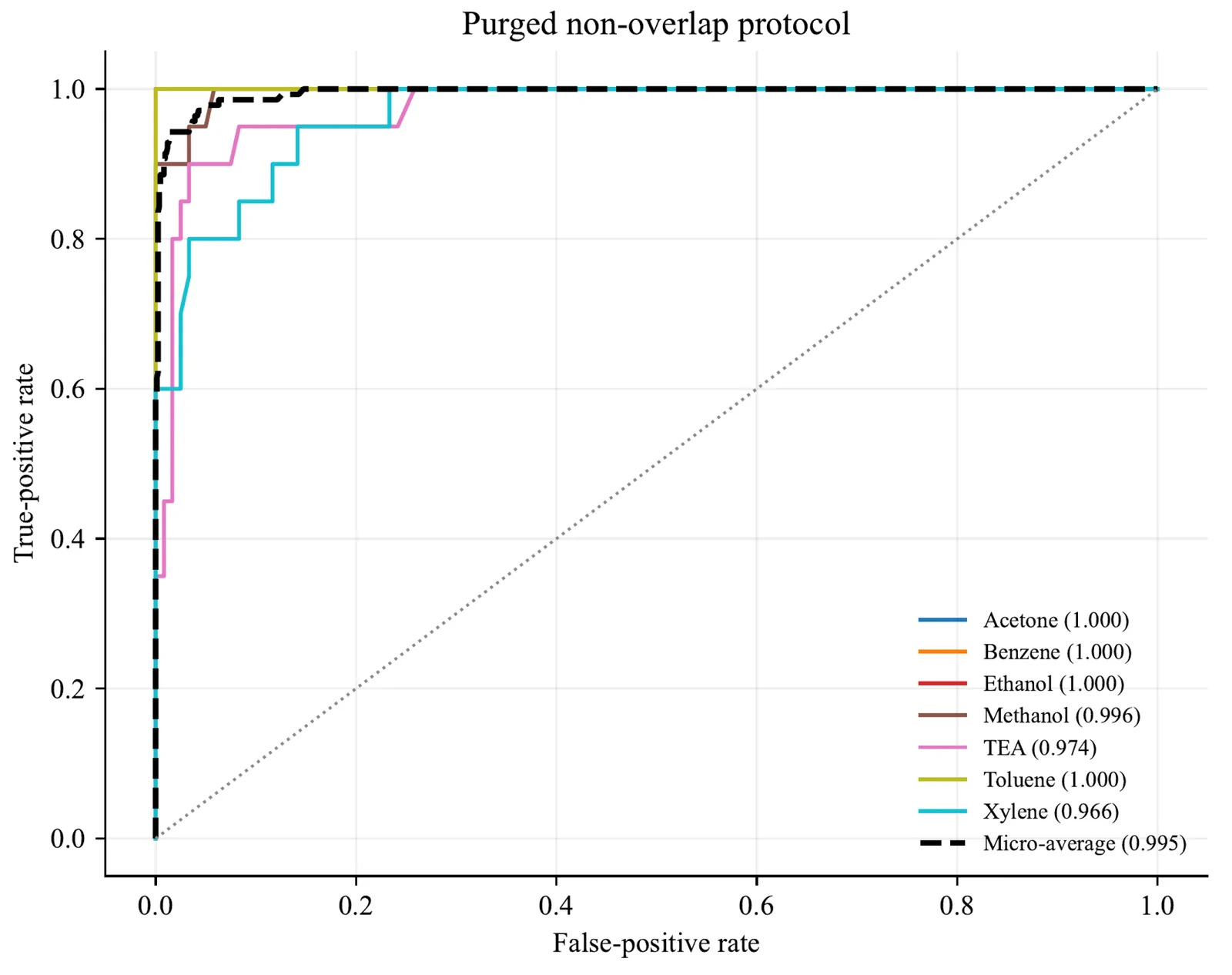
One-vs-rest ROC curves for the primary purged non-overlapping test protocol.

One-vs-rest ROC analysis produces a micro-average AUC of 0.9948 (99.48%). This value measures probability ranking over all decision thresholds and, therefore, should not be interpreted as the 92.14% top-1 classification accuracy. The AUC values for acetone, benzene, ethanol, and toluene are 1.000, while methanol, TEA, and xylene achieve 0.9956, 0.9740, and 0.9656, respectively. The relatively lower AUC values for TEA and xylene are consistent with their residual confusion and partial proximity in Figure 7. Figure 8 shows the corresponding one-vs-rest ROC curves.

The ROC curves provide a separate probability-ranking view: the isolated classes achieve perfect or near-perfect AUC, while TEA and xylene have the lowest class-specific AUC values, consistent with the errors identified by Figure 7.

Taken together, the strict temporal split, feature ablation, classifier controls, overlap sensitivity, seed analysis, t-SNE visualization, confusion matrix, and ROC analysis show that the MsDL+Corr representation retains useful discrimination under a substantially more conservative window-level evaluation than the legacy protocol. Because the source dataset does not retain independent exposure-cycle identifiers, these results quantify temporally separated within-record classification rather than generalization across independently repeated exposure experiments.

## 5. Conclusions

This paper proposed a multi-scale temporal-dependency-learning framework for single-sensor gas classification. The framework combines correlation-based statistical-dependency features and MsDL-based dynamic dependency features to characterize local variations and longer-term evolution in continuously acquired MOS sensor responses.

The validation procedure was reconstructed by partitioning each raw gas-response record chronologically before window extraction and excluding 2000 samples between the training, validation, and test blocks. Under the primary non-overlapping protocol, MsDL+Corr with RF achieved an accuracy of 92.14% and a macro-F1 score of 92.08%, with a micro-average AUC of 0.9948. Feature ablation showed that MsDL provided the main contribution and that correlation features increased macro-F1 by a further 0.86 percentage points. Across ten RF seeds, test accuracy was 92.43% ± 0.37%, indicating limited sensitivity to RF initialization.

The stricter validation reduced accuracy by 3.21 percentage points relative to the legacy window-first protocol, demonstrating that window construction and temporal partitioning materially affect the reported performance. Residual classification errors were concentrated mainly between TEA and xylene. LR slightly exceeded RF on the held-out block, so RF should be regarded as the prespecified primary classifier rather than an unequivocally superior classifier.

Several limitations remain. The available processed dataset does not retain identifiers for independently repeated exposure cycles; therefore, the present evaluation measures classification across temporally separated blocks from the same continuous records rather than generalization across independent experiments. In addition, the experiments use one sensor, pure gases at a fixed concentration of 20 ppm, and controlled laboratory conditions. Performance under different concentrations, mixtures, interfering gases, humidity levels, sensor drift, devices, and acquisition batches remains unverified. Future work should collect explicitly indexed independent exposure cycles and evaluate cycle-disjoint, concentration-disjoint, cross-day, and cross-device generalization.

## Figures and Tables

**Table 1:** Dataset construction and chronological subset definition.

Property	Value
Dataset Type	Continuous single-sensor response records
Gas Categories	7
Sequence Length per Gas	150,000 samples
Training Raw Range	0–87,999
Validation Raw Range	90,000–117,999
Test Raw Range	120,000–149,999
Purge Interval	2000 samples
Primary Window/Stride	1500/1500 samples
Training/Validation/Test Windows	406/126/140

**Table 2:** Main feature extraction and classification parameters.

Parameter	Value
Reservoir Dimension	50
Input Channels	Response, first difference, second difference
Input Scale	0.5
Ridge Coefficient	1.0
Temporal Skips	1,2,4,8,16,32
MsDL Features	906
Correlation Features	12
Combined Features	918
Primary Classifier	RF, 200 trees
Random Seed	42

**Table 3:** Test performance under different temporal validation protocols.

Protocol	Test Windows	Accuracy (%)	Macro-F1 (%)
Legacy window-first	560	95.36	95.31
Purged, 50% overlap	273	94.51	94.44
Purged, non-overlap	140	92.14	92.08

**Table 4:** Window-size sensitivity under the purged non-overlapping protocol.

Window	Validation Acc. (%)	Validation F1 (%)	Test Acc. (%)	Test F1 (%)
500	83.16	83.16	82.86	82.83
750	90.35	90.36	84.64	84.59
1000	88.27	88.17	84.76	84.50
1250	93.51	93.46	88.10	88.08
1500	97.62	97.64	92.14	92.08
1750	94.64	94.54	85.71	85.33
2000	93.88	93.91	87.62	87.05

**Table 5:** Feature-ablation results on the strict test subset.

Feature Representation	Accuracy (%)	Macro-Precision (%)	Macro-Recall (%)	Macro-F1 (%)
Correlation	82.86	83.31	82.86	82.79
MsDL	91.43	91.39	91.43	91.21
MsDL + Correlation	92.14	92.43	92.14	92.08

**Table 6:** Classifier performance on the strict test subset.

Classifier	Accuracy (%)	Macro-Precision (%)	Macro-Recall (%)	Macro-F1 (%)
RF	92.14	92.43	92.14	92.08
SVM	82.86	83.95	82.86	82.60
LR	92.86	93.18	92.86	92.92
KNN	82.14	82.26	82.14	81.57

**Table 7:** Sensitivity to within-subset window overlap.

Overlap	Stride	Test Windows	Accuracy (%)	Macro-F1 (%)
0%	1500	140	92.14	92.08
25%	1125	182	93.96	93.88
50%	750	273	94.51	94.44

## Data Availability

The data presented in this study are available on request from the corresponding author.
